# Oxidative stress in malaria and typhoid fever: A scoping review of the pathogenic mechanisms and therapeutic implications

**DOI:** 10.1371/journal.pgph.0005193

**Published:** 2025-10-09

**Authors:** Palmer Masumbe Netongo, MacDonald Bin Eric, Severin Donald Kamdem, Ange Maxime Tchoutang, Wilfred Fon Mbacham

**Affiliations:** 1 Department of Biochemistry, University of Yaoundé 1, Yaoundé, Cameroon; 2 Molecular Diagnostics Research Group, Biotechnology Centre, University of Yaoundé 1, Yaoundé, Cameroon; 3 Laboratory for Public Health Research Biotechnologies, Biotechnology Centre, University of Yaoundé 1, Yaoundé, Cameroon; 4 School of Science, Navajo Technical University, Crownpoint, New Mexico, United States of America; 5 Department of Pathology, University of Utah School of Medicine, Salt Lake City, Utah, United States of America; 6 School of Health Sciences, Catholic University of Central Africa, Yaoundé, Cameroon; Child Health Research Foundation, BANGLADESH

## Abstract

Oxidative stress is a key factor in the pathogenesis of many infectious diseases, including typhoid fever and malaria. Co-infection with these diseases poses a particular challenge, as both are associated with increased production of reactive species that can damage tissues, trigger oxidative stress, and exacerbate disease. To cope with this oxidative insult, cells have a defense system consisting of antioxidant molecules that can terminate the oxygen radical cascade and render toxic metabolites innocuous. However, lack of complete understanding of the pathophysiology of malaria and typhoid fever continues to hinder efforts aimed at eradicating these diseases. In this review, we examine the current understanding of oxidative stress in the context of malaria and typhoid fever, and its implications for disease pathogenesis and treatment. The search and data extraction strategy used in this scoping review involved a structured approach to identify relevant studies related to the topic. Studies were included that investigated ROS production, antioxidant responses, and the effect of pharmacological interventions on oxidative stress markers. The search was conducted using electronic databases, including PubMed, ScienceDirect, and Directory of Open Access Journals (DOAJ), and involved an extensive literature search of studies published between January 2000 and May 2024. The review identified potential avenues for research with evidence that both malaria and typhoid fever elevate oxidative stress levels, which are further exacerbated in co-infections. The endogenous antioxidant response is activated but may not be sufficient to neutralize ROS completely. Moreover, commonly used antimalarial and antibacterial drugs influence oxidative and antioxidant dynamics, which may have implications for treatment efficacy and disease progression. Understanding the oxidative-antioxidant balance in malaria and/or typhoid fever may open new avenues for adjunctive therapies and vaccines targeting oxidative stress. It is thus important to consider the oxidative-antioxidant balance during treatment of malaria and typhoid fever patients.

## 1. Introduction

Malaria and typhoid fever are among the most significant infectious diseases impacting public health in tropical regions, particularly in sub-Saharan Africa and South Asia. These diseases often coexist due to overlapping environmental and socioeconomic determinants and accumulating evidence suggests that malaria is a risk factor for salmonella infection. Their comorbidity poses unique diagnostic and treatment challenges, compounded by similar clinical symptoms and the potential for synergistic pathophysiological effects. A growing body of evidence implicates oxidative stress as a central mechanism in the pathogenesis and severity of both malaria and typhoid fever, as well as in their treatment outcomes [1–4].

Although the rates of both infections have decreased in some countries, resistance to drug therapy has increased, particularly in patients infected with *Plasmodium falciparum* and *Salmonella typhi*, which are responsible for the majority of cases in Africa. The factors leading to this resistance are not yet well understood, owing to a lack of comprehensive knowledge regarding the pathophysiological mechanisms of both diseases, especially in conjunction. A number of studies have discussed the implications of free radicals and oxidative stress in the pathophysiology of malaria and typhoid fever [5–12]. This involvement may be related to the pathogenic mechanisms triggered by the pathogen in question as well as free radical production and antioxidant defenses in host cells to minimize the effect of the infection [13–15]. However, the ultimate role of oxidative stress during infection remains unclear. Some authors suggest a protective role, while others propose a link to the pathophysiology of the disease [6,16,17].

Despite the substantial burden of these diseases, current treatment protocols largely overlook the contribution of redox imbalance to disease progression and recovery. Investigating oxidative stress not only offers insights into disease mechanisms but also opens avenues for adjunctive antioxidant therapies to improve clinical outcomes. Thus, a better understanding of the pathophysiological mechanisms and the host response during such co-infections is necessary. In this scoping review, we critically synthesise current evidence on oxidative stress mechanisms in malaria and typhoid fever, evaluate the impact of therapeutic agents on redox balance, and identify gaps for future research.

## 2. Methodology

### 2.1. Design and search strategy

This scoping review was conducted in accordance with the PRISMA Extension for Scoping Reviews (PRISMA-ScR) to systematically map existing evidence on oxidative stress markers in malaria, typhoid fever, and co-infection, with a focus on disease pathogenesis and therapeutic implications (S1 Data).

**Fig 1:**
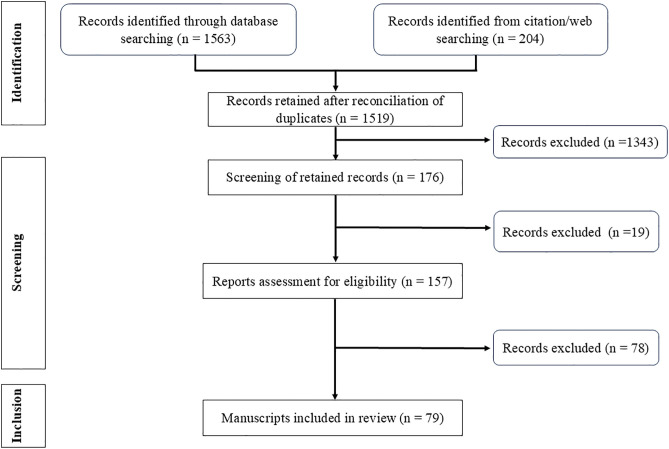
PRISMA flowchart for study identification and screening.

The search strategy involved a comprehensive and structured approach to identify relevant studies related to the topic (S1 File). The search was conducted using electronic databases, including PubMed, ScienceDirect, and Directory of Open Access Journals (DOAJ), and involved an extensive literature search of studies published between January 2000 and May 2024. Manuscripts identified, screened and cited followed the outline in Fig 1. The search strategy was based on relevant keywords such as “oxidative stress,” “malaria,” “typhoid fever,” “pathogenesis,” “*Plasmodium* infection,” “*Salmonella* infection,” “treatment,” and “antioxidant.”

### 2.2. Eligibility criteria and study selection

We included studies that reported on oxidative stress and its role in the pathophysiology and treatment of malaria and/or typhoid fever infections in both humans and animal models. The inclusion criteria for the studies in this review paper were: **(i)** original and review articles published in peer-reviewed journals from January 2000 to May 2024 in English language only, **(ii)** were conducted in human, animal, or in vitro/in vivo models, **(iii)** studies that reported on the relationship between oxidative stress and malaria or typhoid fever, and **(iv)** studies that examined the pathophysiology of oxidative stress in malaria and typhoid fever. However, we excluded case reports, editorials, commentaries, conference abstracts, or non-original studies unless comprehensive reviews.

We used the **‘OR,’ ‘AND’** and **‘NOT’** Boolean logical operators in search queries (S2 File) to refine the search terms and narrow down the results. For the purpose of this review, we use the following combination of the keywords with “OR” within each concept and with “AND” between the concepts:

Search: (((((**Typhoid fever** OR ***Salmonella* typhi Infection** OR **Abdominal Typhus** OR **Enteric Fever**) **AND** (**Oxidative stress** OR **Stress** OR **Oxidative DNA Damage** OR **Oxidative Damage** OR **Nitrosative Stress** OR **Nitro-Oxidative Stress** OR **Oxidative Nitrative Stress** OR **Oxidative Injury** OR **Oxidative Stress Injury** OR **Oxidative Cleavage** OR **Antioxidative Stress** OR **Anti oxidative Stress**)) OR (**Antioxidant** OR **Endogenous Antioxidants**)) **AND** ((**Malaria** OR ***Plasmodium* Infection** OR **Paludism** OR **Remittent Fever** OR **Marsh Fever**) **AND** (**Oxidative stress** OR **Stress** OR **Oxidative DNA Damage** OR **Oxidative Damage** OR **Nitrosative Stress** OR **Nitro-Oxidative Stress** OR **Oxidative Nitrative Stress** OR **Oxidative Injury** OR **Oxidative Stress Injury** OR **Oxidative Cleavage** OR **Antioxidative Stress** OR **Anti oxidative Stress**))) **AND** (***T*herapy** OR **Therapeutic** OR **Treatment**)) **AND** (**Pathogenesis** OR **Pathogeny** OR **Pathogenicity** OR **Pathology**) **Filters:** English, Exclude preprints, from January 2000 – May 2024.

### 2.3. Screening, data extraction and synthesis

All retrieved records were imported into a Mendeley citation management tool (version 1.19.8), and duplicates were removed. Three reviewers independently screened the studies based on the inclusion and exclusion criteria and full-text review of the articles obtained from the databases. Disagreements between reviewers were resolved through discussion and consensus; if consensus could not be reached, a fourth reviewer was consulted.

Data were charted using a standardized form developed for this review. The following information was extracted from each study: author(s), year of publication, study location, sample size and population characteristics, type of infection studied (malaria, typhoid, or co-infection), oxidative and antioxidant biomarkers measured, key findings, and relevance to pathogenesis or treatment. Studies evaluating drug treatments (e.g., antimalarials or antibiotics) and their relationship to oxidative stress or antioxidant capacity were specifically flagged and analyzed separately.

Results were narratively synthesized and organized thematically into categories: [1] oxidative stress in malaria; [2] oxidative stress in typhoid fever; [3] oxidative stress in malaria–typhoid co-infection; and [4] the impact of antimalarial and/or antibiotic treatment on oxidative/antioxidant markers. Key findings were tabulated to illustrate patterns across study types and populations. Given the heterogeneity of methodologies and outcomes, no meta-analysis was conducted.

### 2.4. Ethical approval

Since the present review paper is based exclusively on already published studies and does not involve any primary data collection, formal ethical approval may not be necessary. However, we have ensured that our literature search, data extraction, and analysis have been carried out in an ethical and transparent manner. We have followed the established ethical guidelines and principles for conducting reviews, including searching multiple databases, selecting relevant studies based on pre-specified criteria, and evaluating the quality of the included studies. We have also made sure to accurately and responsibly report the results of our analysis without misleading or misinterpreting the information. Therefore, we assure the readers that this review has been carried out in accordance with ethical standards and integrity.

## 3. Results

In this scoping review, we systematically mapped evidence from over two decades of research on oxidative stress and antioxidant responses in malaria, typhoid fever, and their co-infection. Several studies (see Table 1) have revealed that ROS generation and impaired antioxidant defenses are consistently implicated in malaria and typhoid fever pathogenesis, severity and treatment outcome.

**Table 1:** Summary of key studies assessing oxidative stress indicators and drug modulation in malaria, typhoid, and co-infections.

SN	Reference	Sample size	Age range (yrs)	Study population	Major biomarkers	Major findings and comments
1	Abubakar *et al*., 2016 [23]	160	1 – 10	*Pf* infected and uninfected children	MDA, Vit A, C, & E; and GSH	↓ Antioxidant (p < 0.05) ↑ MDA in patients
2	Kamble *et al*., 2011 [24]	60	15 – 60	*Pf* and *Pv* infected, and healthy individuals	MDA and serum ceruloplasmin	↑ MDA, ↑ ceruloplasmin (p < 0.001) in *P. falciparum* than *P. vivax* and controls
3	Krishna *et al*., 2009 [25]	110	15 – 55	Patients diagnosed for different types of malarial infections	Total cholesterol, LDL, HDL, triglyceride, MDA	↑ Total cholesterol, ↑ LDL, ↑ triglycerides and ↑ MDA, ↓ HDL patients compared to controls
4	Nidhi et al., 2012 [26]	100	0 – 15	Cases of severe malaria + control	MDA, protein carbonyl, nitrite, Vit C and Cu, SOD, GSH, ceruloplasmin	↓ ceruloplasmin, ↓ GSH and ↓ SOD in SM, ↑ MDA and ↑ protein carbonyl (p < 0.05)
5	Olusegun MA., 2015 [27]	170	0 – 5	Children with fever, diarrhoea, headache, vomiting, and some other malaria symptoms	Triglyceride, total plasma protein, MDA, SOD and GSH	↑ Total protein, ↑ SOD, ↑ GSH in 0–1 yr. ↑ triglyceride, ↑ MDA in 2–5 years (p < 0.05).
6	Ezzi et al., 2017 [28]	90	18 – 48	Symptomatic patients	Ceruloplasmin (CP), Vit C, SOD, ALP and Lactate dehydrogenase (LDH)	↑ CP (p < 0.001), ↓ SOD, ↓ Vit C, ↑ LDH and ↑ ALP (p < 0.0001)
7	Shukla et al., 2009 [29]	48	8-10 weeks,	Crossbred LACA mice, 18-25g	SOD, CAT; MDA, GSH	↑ MDA, ↑ GSH, ↓ SOD, ↓ CAT in all organs of co-infected mice compared to control
8	Benedicta et al., 2009 [30]	50	18 – 60	Untreated *P. vivax* and *P. falciparum* malaria patients + healthy control	SOD, CAT; MDA	↓ SOD, ↓ CAT and ↑ MDA (p < 0.001)
9	Oluba *et al.,* 2014 [31]	NA	NA	Mice	MDA, SOD, GPx, GST, G6PDH	↓ MDA, ↑ SOD, ↑ GPx, ↑ GST, ↑ G6PDH (p < 0.05) in treated mice compared to infected but untreated group.
10	Mahamat et al., 2020 [32]	300	NA	Pregnant women suspected of having malaria	NA	Low total oxidative stress (TOS), and antioxidant defence (TAD) of cord blood
11	Nsiah et al., 2019 [33]		< 12	Patients with symptoms of malaria	MDA, Vit C	↑ MDA levels and ↓ Vit C
12	Bayim et al., 2012 [12]	86	NA	Treated and untreated typhoid fever patients	MDA, Vit C, β– carotene and total cholesterol	↓ MDA, ↑ total cholesterol, ↑ β– carotene and ↑ Vit C during and after treatment compared to untreated group.
13	Garba et al., 2011 [34]	40	15 - 45	Patients with typhoid perforation before and after surgical intervention	Antioxidant metalloenzymes (SOD, CAT); MDA, redox-active metals (Cu, Zn, Fe, Mn)	↑ MDA in pre-surgical period, ↓ CAT and ↓ SOD with elevated ↑ Cu and ↑ Zn
14	K.H. Khan, 2009 [35]	58	5-7 weeks	Mice pretreated with T500 for a period of 30 days	MDA, CAT and GSH	Infected mice showed ↑ GSH by 62% and ↓ of 5.6% in CAT and 97.69% ↓ LPO
15	S.S.Haque, 2011 [15]	NA	6-8 weeks old mice	Infected mice	Catalase, Xanthine Oxidase and nitric oxide precursor	↑ XO and ↑ NO with ↓ CAT activity
16	Ezeigbo and Nwaehujor, 2010 [36]	152	Varying ages	Clinically confirmed typhoid patients	Total lipid, total cholesterol and MDA	↑ MDA and ↓ cholesterol in patients compared to control
17	Patel et al., 2018 [37]	06	NA	BALB/c mice co-infected malaria + *Salmonella*	MDA, GSH, catalase, SOD	Co-infection → ↑oxidative stress + liver damage; artesunate restored balance
18	Siddiqi et al., 2002 [38]	NA	NA	P. yoelii mice models	Lipid peroxidation, catalase, SOD	CQ altered antioxidant defense in malaria
19	Singh et al., 2022 [39]	NA	NA	Mice vaccinated with Salmonella OMPs	ROS, free radicals	↑ GSH in liver, kidney, and lungs, ↓ CAT in kidney and lungs of vaccinated mice

### 3.1. Origin of oxidative stress during plasmodium infection

One important aspect of malaria pathophysiology is its impact on hemoglobin metabolism and oxidative stress [18,19]. When *Plasmodium* infects red blood cells, it begins to break down hemoglobin to obtain amino acids for its survival. This process results in the release of heme, a toxic byproduct that can induce oxidative stress and damage both the parasite and the host cell. Prolonged exposure to high levels of heme and other ROS can overwhelm cellular defenses, leading to increased oxidative stress and damage [18]. This contributes to the progression of the disease and its complications, such as anemia, cerebral malaria, and organ failure.

In response to infection caused by *Plasmodium* parasites, the natural host defense system is activated, involving phagocytes (macrophages and neutrophils). As a result, these phagocytes generate a substantial amount of reactive oxygen and nitrogen species, leading to a disturbance in the activities of both oxidant and antioxidant species. This imbalance triggers oxidative stress, which is an important mechanism of response by human hosts to infections. In the case of malaria, this oxidative stress can lead to the death of the parasites [20,21].

Systemic hyper-activation in *Plasmodium*-infected blood cells is characterized by elevated levels of circulating nitrogen oxide reactive intermediates [22]. During the blood stage of the *Plasmodium* life cycle, there are various sources of host-generated oxidative stress, including direct effects from erythrocyte invasion, heme degradation, and the host’s response to infection. These factors contribute to the systemic upregulation of oxidative enzymes and the phagocytic oxidative burst. As the malaria parasites grow and multiply rapidly, their metabolic rates increase proportionately, resulting in the generation of large quantities of toxic oxidant by-products (reactive oxygen species and reactive nitrogen species). This process ultimately leads to the degradation of host hemoglobin. Consequently, the detoxification of these reactive species poses a significant challenge for erythrocytes infected with *Plasmodium*.

#### 3.1.1. Haemoglobin metabolism and oxidative stress.

Hemoglobin metabolism is a complex process that involves breaking down hemoglobin into its constituent parts: heme and globin. Heme is further metabolized into biliverdin, which is eventually converted to bilirubin and eliminated from the body via the liver. ROS are highly reactive molecules that can damage cells and tissues if not adequately controlled. The body employs various antioxidant defense mechanisms to counteract oxidative stress; however, excessive ROS production can overwhelm these defenses, leading to cellular damage and disease. In various pathologies, such as malaria and other hemolytic diseases, large quantities of hemoproteins are released into the plasma [40,41]. Hemoglobin, which comprises approximately 95% of the proteins in red blood cell cytosol, has an estimated concentration of 340 mg/ml [42]. It serves as the primary nutrient source for the parasite. Once a red blood cell is invaded, the parasite targets and degrades hemoglobin, with *Plasmodium falciparum* consuming an estimated 75% of the hemoglobin during its brief intra-erythrocytic life cycle [43].

**Fig 2:**
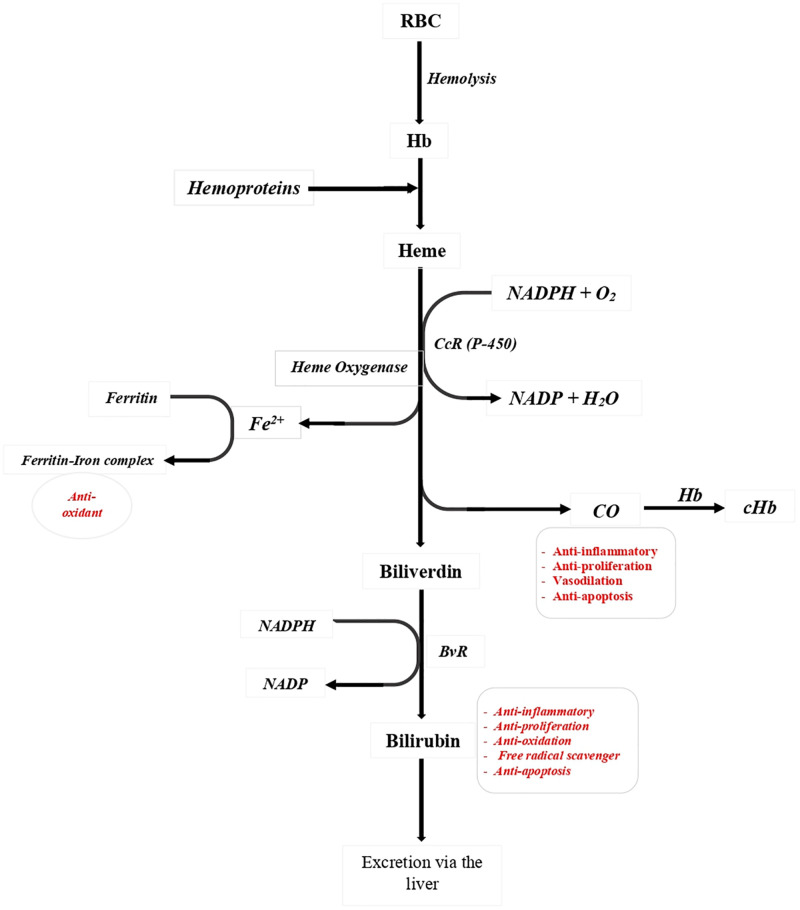
Degradation of heme by host and the formation of bilirubin. Heme released from the haemoglobin (Hb) of red cells (RBC) or from other hemoproteins is degraded by an enzymatic process involving heme oxygenase (HO), the first and rate-limiting enzyme in a two-step reaction requiring cytochrome reductase p-450 (CcR) NADPH and oxygen, and resulting in the release of iron (Fe^2+^) and the formation of carbon monoxide (CO) and Biliverdin (Bv). The CO could exert an anti-inflammatory, anti-proliferation, vasodilation, anti-apoptosis in the immune system. Biliverdin is further reduced to bilirubin (BR) by biliverdin reductase (BvR). In addition to the role of CO, BR could play the role of anti-oxidation, free radical scavenger. Figure is adapted from Haines & Tosaki, 2020 [44] and Wu et al., 2019 [41].

During the ring stage of its lifecycle, *Plasmodium* exhibits some detectable levels of hemoglobin degradation; however, the majority of this metabolic process occurs during the trophozoite and schizont stages. The trophozoite has limited metabolic capacity and breaks down hemoglobin incompletely due to the absence of the enzyme heme oxygenase, which vertebrates utilize for heme catabolism. This breakdown releases heme, which is highly toxic to the parasite. Hemoglobin degradation takes place within the parasite’s acidic food vacuole, a critical process for its survival within the host erythrocyte. This degradation serves as the primary source of fuel and amino acids for the parasite, as it has a limited ability to synthesize its own amino acids. This catabolic activity also produces ROS and free heme (ferri/ferroprotoporphyrin IX; FP), which can be toxic to both the parasite and the red blood cells (Fig 2) [44].

The red blood cell, which serves as the habitat for the parasites, can be considered a pro-oxidant environment due to the presence of oxygen and iron. These elements are essential for the formation of RO) through the Fenton reaction (see Fig 2). When parasites ingest hemoglobin into their acidic food vacuole, this process is accompanied by the spontaneous oxidation of Fe²⁺ to Fe³⁺ and the generation of superoxide anions. To mitigate this toxicity, the parasite sequesters free protoporphyrin (FP) into intracellular aggregates of insoluble heme crystals known as hemozoin (malaria pigment), where heme molecules become interlinked (Fig 2) [41,44,45]. Hemozoin is non-toxic and does not induce oxidative damage to either the host or the parasites. However, since not all heme molecules can be effectively crystallized into hemozoin within the infected erythrocyte, free heme can still potentially harm host cells and tissues. Additionally, when uninfected red blood cells undergo lysis, heme molecules are released into the bloodstream.

Research has shown that the lysis of both infected and uninfected erythrocytes can contribute to oxidative stress in malaria [46]. In addition to obtaining nutrients and amino acids from hemoglobin degradation, the parasite digests erythrocyte hemoglobin to prevent premature lysis, which could occur if the parasite fails to manage the increased cell volume. Ultimately, hemoglobin metabolism can lead to oxidative stress, suggesting that antioxidant interventions may be beneficial for mitigating this stress and improving overall health outcomes.

**Fig 3:**
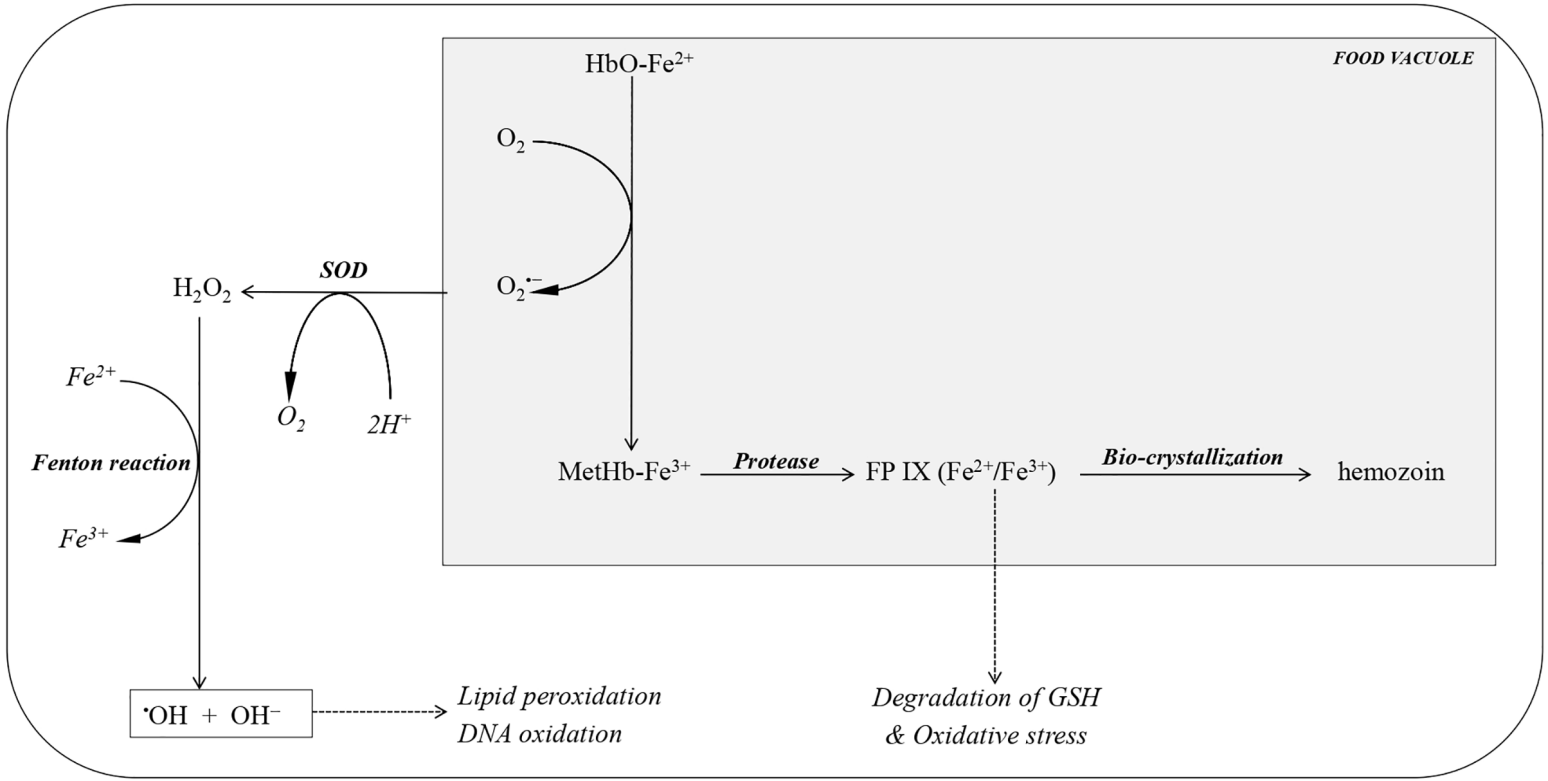
Origin of reactive oxygen species during *plasmodium* infection. FP IX, ferri/ferroprotoporphyrin IX; HbO-Fe2 + , oxy-haemoglobin containing ferriprotoporphyrin IX; MetHb-Fe3 + , methaemoglobin containing ferro-protoporhpyrin IX; SOD, superoxide dismutase. Digestion of haemoglobin in the parasite’s food vacuole during malaria infection is the chief source of ROS and oxidative stress. Most of the free haem (FP IX) released from the digested haemoglobin is converted to hemozoin within the parasite’s food vacuole. However, some of the FP IX is released from the vacuole into the cytosol where it undergoes oxido-reduction reactions leading to the generation of superoxide anions, degradation of GSH and oxidative stress within the parasite. Superoxide anions, O_2_^•−^ resulting from the oxidation of haem-iron, HbO-Fe^2+^ in haemoglobin, are either detoxified by SOD, to yield H_2_O_2_, or can react in a spontaneous reaction with H_2_O_2_ leading to the formation of hydroxyl radicals in the presence of free iron via the Fenton reaction. These are highly reactive radicals that can trigger lipid peroxidation and DNA oxidation. The malaria parasite lacks catalase and glutathione peroxidases which can readily detoxify H_2_O by reduction so this is exclusively achieved using thioredoxin peroxidases.

The endocytosis of hemoglobin into the parasite’s food vacuoles induces the spontaneous oxidation of heme-iron from its ferrous form (Hb-Fe²⁺) to the ferric form (MetHb-Fe³⁺), also known as hemin. This process subsequently triggers the formation of superoxide radicals (O₂•⁻). The reaction between hemin and superoxide radicals leads to the production of hydrogen peroxide (H₂O₂) and, ultimately, hydroxyl radicals (OH•⁻), which are highly reactive and cytotoxic species (Fig 3).

#### 3.1.2. Oxidative changes in *Plasmodium* organism.

ROS during parasitic infections primarily arise from both the metabolism of the parasite and the host’s immune response [6]. In erythrocytic parasites, two significant sources of reactive free radicals include the mitochondrial electron transport chain and the degradation of hemoglobin [21]. The parasite employs its antioxidant machinery and detoxifies the heme produced during hemoglobin digestion as key mechanisms for maintaining redox homeostasis [6]. In addition to ROS generated by the host in response to *Plasmodium* infection, the parasite itself can directly secrete highly reactive oxygen species, which disrupt biochemical reactions occurring within red blood cells. This secretion also aids in the parasite’s survival and integration within liver and blood cells [46]. Furthermore, it has been proposed that aerobic membrane transport mechanisms are a major source of ROS generation in *Plasmodium* [47].

#### 3.1.3. Antioxidant defense system in the malaria parasite.

The host and the malaria parasites both encounter high levels of oxidative stress during the development of the parasite within host cells, making their ability to defend against this stress crucial for survival. To mitigate oxidative damage, the parasite employs several antioxidant defense mechanisms. Gene expression profiling studies indicate that, during the erythrocytic developmental stage, a continuous cascade of genes is activated to coordinate the parasite’s antioxidant responses under these conditions [48–50]. Additionally, to reduce the harmful effects of oxidative stress, the parasite regulates the production of reactive species and adapts to the host’s antioxidant defenses. It has been suggested that the parasite utilizes its apicoplast as a vital organelle for survival, contributing significantly to lipid metabolism [51]. The apicoplast, an intracellular symbiotic organelle located near the mitochondria, is responsible for synthesizing lipoic acid—a potent antioxidant that the parasite employs as a defense mechanism [21].

In *Plasmodium* cells, the glutathione and thioredoxin redox systems serve as two powerful mechanisms for detoxifying reactive oxygen species, particularly in *Plasmodium falciparum* malaria. These systems effectively help prevent parasite development within host cells [52]. It has also been hypothesized that the parasite utilizes a chromatin-associated enzyme called peroxiredoxin, which employs thioredoxin and glutaredoxin as reducing agents to protect against oxidative damage inflicted by the host [21,53,54]. Additionally, a study has demonstrated the critical role of α-tocopherol in the defense mechanisms of *Plasmodium falciparum* against environmental stress, including its capacity to maintain ROS levels. The parasite synthesizes α-tocopherol, which exists in its oxidized form as a protective measure against oxidative stress. Notably, this antioxidant may also be utilized by the host for similar protective purposes [16].

#### 3.1.4. Oxidative stress as host defense mechanism against *Plasmodium* infection.

**Fig 4:**
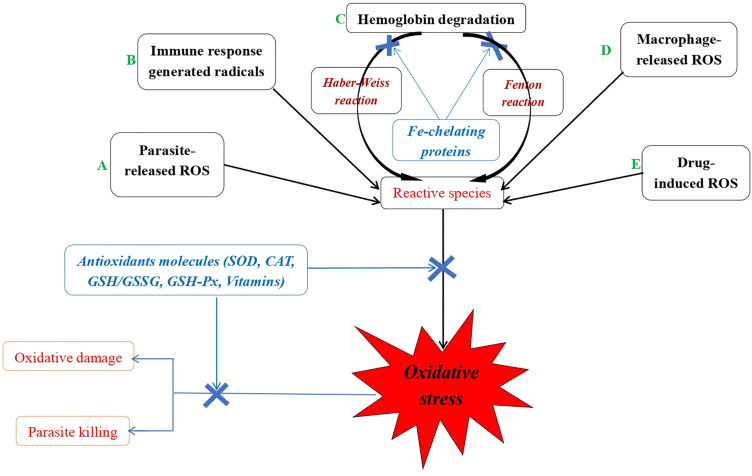
Sources of reactive oxygen species, ROS (*A, B, C, D, E*) and antioxidant mechanism system (blue) during malarial infection. SOD = superoxide dismutase; CAT = catalase; GSH-Px = glutathione peroxidase; GSH = reduced form of glutathione; GR = glutathione reductase; GSSG = oxidized form of glutathione.

In healthy individuals, the production of reactive species is regulated by both enzymatic and non-enzymatic antioxidant defense systems [55]. Antioxidant substances react with reactive free radical species, donating electrons and chemically stabilizing these radicals without generating reactive metabolites. The enzymatic antioxidant system is mediated by a group of biological catalysts. In response to oxidative stress, most cells produce five major antioxidant enzymes: SOD, CAT, glutathione peroxidase (GPx), glutathione S-transferases (GST), and peroxiredoxins (Prx) [56]. These antioxidant enzymes can be categorized into three systems: the first includes SOD and CAT, which provide the first line of defense against superoxide and hydrogen peroxide; the second comprises the thioredoxin-independent system, which includes Prx and utilizes electrons supplied by the thiol groups of thioredoxins to reduce their targets; and the third system involves GST, which obtains electrons from GSH. Together, these antioxidant molecules help minimize cell damage caused by free radicals and are essential for maintaining optimal health. Additionally, they are known to regulate immune responses by stimulating or inhibiting the production of specific cytokines, influencing transcription factors, and regulating cellular processes such as apoptosis (Fig 4).

SOD is a metalloenzyme that catalyzes the dismutation of superoxide ions (a type of reactive species) into hydrogen peroxide, which is less reactive. This enzyme plays a crucial role in the antioxidant systems of various tissues exposed to ROS [21,55]. The essential function of SOD activity in providing anti-parasitic defense during *P. falciparum* malaria infections in children was recently reported, with evidence indicating that this activity may lead to damage in the parasite’s DNA [57].

Another key player in the cellular antioxidant defense system is CAT, which belongs to the subclass of enzymes known as oxidoreductases. Catalase is primarily located in the cytoplasm and nucleus of red blood cells and liver cells and is directly involved in metabolizing the high concentrations of hydrogen peroxide that are produced by SOD. By doing so, CAT helps prevent the formation of hydroxyl radicals (•OH), which can damage membrane components. The activity of catalase increases in response to high levels of hydrogen peroxide, while lower concentrations are regulated by the glutathione system [58].

In addition to antioxidant enzymes, reduced GSH is the primary antioxidant molecule produced by cells. GSH plays a vital role in neutralizing reactive species and protecting cells from injuries resulting from lipid and protein peroxidation. Within the cells, glutathione exists in three forms: reduced (monomeric, or GSH), oxidized (dimeric, or GSSG), and conjugated (GS-R), with the reduced form being the most prevalent in healthy tissues [55]. The glutathione system works synergistically with the enzymatic antioxidant system, which includes glutathione peroxidase and glutathione S-transferase (GST), to defend against ROS. Furthermore, glutathione is involved in regulating immune responses to various infectious etiologies, including malaria, and may offer potential protective effects against the disease’s progression [55,59].

A substantial and growing body of literature has elucidated the role of other molecules like the nuclear factor erythroid 2-related factor 2 (Nrf2) signalling in the host’s defense mechanisms, particularly in the context of combating oxidative stress induced by parasitic infections. Nrf2 is a transcription factor that regulates the expression of various antioxidant genes and is pivotal in maintaining cellular redox homeostasis [60,61]. Under conditions of oxidative stress, such as that caused by the host’s immune response to parasites, Nrf2 is activated and it translocates to the nucleus, where it binds to antioxidant response elements (AREs) to drive the expression of a variety of protective genes. Research has demonstrated that Nrf2 activation serves to counteract oxidative damage by upregulating the expression of enzymes involved in detoxifying ROS, such as heme oxygenase-1 (HO-1), SOD and GPX [61–63].

#### 3.1.5. Anti-malarial drugs as potential source of oxidative stress.

Many substances used as antimalarials are pro-oxidants, which contributes to their pharmacological efficacy. Examples include chloroquine, primaquine, and artemisinin, among others. Their pro-oxidant effect may arise from the drugs’ ability to promote the direct production of free radicals or to inhibit molecules with antioxidant activity. Research has shown that the malaria parasite is highly sensitive to ROS-mediated oxidative stress. This sensitivity allows various antimalarial treatments to exploit this vulnerability to inhibit the parasite’s growth and proliferation within human hosts. A notable example is quinolines, which act as blood schizonticides. By interfering with the heme detoxification process, they generate reactive species that target the parasite. Quinolines have historically been the most commonly used group of antimalarials before the introduction of artemisinin-based combination therapies (ACTs). Drugs like chloroquine (CQ) and amodiaquine (AQ) bind to ferriprotoporphyrin (FP) and inhibit its polymerization into hemozoin, leading to its accumulation in the parasite’s digestive vacuole [21,56,64].

Since some antimalarial drugs may target similar mechanisms and pathways, the varying levels of RO) generation among these drugs could explain their differing pharmacological activities. A well-established mechanism of chloroquine resistance is the parasite’s ability to prevent the accumulation of the drug in the food vacuole. Additionally, an enhanced antioxidant capacity in the parasite may contribute to its resistance. Despite numerous control strategies currently in use or in development, there has been a concerning rise in resistance among *Plasmodium* parasites. This resistance can be attributed to the parasites’ ability to evade the oxidative stress caused by ROS, both under steady-state conditions and during treatment. Studies have shown that chloroquine-resistant strains of malaria parasites exhibit increased activity of the antioxidant enzyme glutathione S-transferase compared to chloroquine-sensitive strains of the same species. This heightened activity may enable these resistant parasites to mitigate the oxidative burden imposed by the drug [6,18,47].

On the other hand, artemisinin is a compound that rapidly eliminates blood-stage parasites, including *Plasmodium falciparum* strains that are resistant to other antimalarial drugs. This drug generates free radicals upon contact with iron, a metal commonly found in the body, particularly within erythrocytes [6,56]. Artemisinin and its derivatives have been found to accumulate in the parasite’s food vacuole forming artemisinin-FP adducts and ROS that trigger membrane damage and eventually parasite death [16,65]. Artemisinin-generated reactive species were reported to inhibit complex-IV (cytochrome-C oxidase) of *Plasmodium* electron transport chain of the malaria parasites [66]. Although the mechanisms of action of most antimalarial drugs are not entirely understood, it is evident that many exert their effects through the production of ROS. The iron-rich environment within the parasite significantly contributes to the rapid generation of these reactive molecules, which can damage the parasite by targeting various components, including cellular membranes, redox systems, and the mitochondrial electron transport chain. This may account for the swift elimination of parasites by artemisinin derivatives. Beside current antimalarial treatments, a recent study [67] identified a variety of small molecules that are capable of modulating the redox balance of the malaria parasite, leading to oxidative stress and impairing crucial cellular functions. In combination with current antimalarial drugs, they exhibited synergistic effects

### 3.2. Origin of oxidative stress during *Salmonella* infection

During a Salmonella infection, including typhoid fever, the bacterial cells produce various virulence factors to evade the host’s immune response. These factors include lipopolysaccharide (LPS), flagellin, and effectors from the Type III secretion system (T3SS), which can induce oxidative stress in host cells. When these virulence factors are detected by the host’s innate immune system, they trigger the activation of phagocytic cells such as macrophages, neutrophils, and dendritic cells. In response, these immune cells generate ROS and RNS as part of the host defense mechanism to eliminate or contain the invading pathogen. These species are produced by various cellular processes such as NADPH oxidase, inducible nitric oxide synthase (iNOS), and mitochondrial electron transport chain. However, their excessive production can cause damage to the host cells, nucleic acids, and proteins as well as the invading *Salmonella* cells leading to inflammation and immune response which promote pathogenesis of *Salmonella* infection and oxidative stress. Additionally, *Salmonella* cells contain enzymes that neutralize ROS and RNS, such as catalase and SOD, which helps them to survive and replicate within the host [68,69]. Therefore, the interplay between the host’s immune system and the *Salmonella* virulence factors causes oxidative stress in both the host and the pathogen during typhoid fever. This oxidative stress contributes to the pathogenesis of the disease and can lead to tissue damage and systemic inflammation. A study by Raffatellu *et al*. in 2008 [70] showed that *S. typhimurium* infection stimulates ROS production in host cells through NADPH oxidase activation. While another study by Chakravortty et al. in 2002 [71] demonstrated that ROS production in macrophages during *Salmonella* infection may be due to the presence of *Salmonella* pathogenicity island (SPI)-2 genes which encode for T3SS that allow bacterial survival and replication within host cells.

#### 3.2.1. Antioxidant defense system of host during *Salmonella* infection.

The antioxidant defense system in typhoid fever includes various enzymes, such as SOD, catalase, and glutathione peroxidase, and antioxidants such as glutathione and vitamins C and E. These components work together to protect the body against oxidative damage caused by ROS produced during infection. Studies have shown that typhoid fever patients have an imbalance between pro-oxidants and antioxidants, leading to increased oxidative stress and damage to cells and tissues. To combat this, the body activates its antioxidant defense system to scavenge ROS and prevent further damage. Levels of SOD, catalase, and glutathione peroxidase have been reported to be significantly lower in typhoid fever patients compared to healthy controls, indicating decreased antioxidant defense [34]. Additionally, the study showed that administration of antioxidants such as vitamin C and E improved antioxidant status and reduced oxidative stress in typhoid fever patients.

Another study reported a significant depletion of erythrocyte GSH, increase in plasma GSH and increased expression of CAT in both plasma and erythrocyte, characterizing the antioxidant response of the subjects. While the highest erythrocyte CAT activity was observed in typhoid-infected individuals [72]. Thus, administration of exogenous glutathione can restore antioxidant status and reduced oxidative stress. It is therefore evident that the antioxidant defense system plays a crucial role in protecting the body against oxidative damage during typhoid fever. Further research is needed to explore the mechanisms behind this system and potential therapeutic interventions to enhance antioxidant defense in typhoid fever patients.

#### 3.2.2. Antioxidant defense system of the *Salmonella* bacteria.

One of the primary strategies employed by *Salmonella* to evade the host immune system is the activation of its antioxidant defense mechanism, the SoxRS regulon, which helps the bacteria cope with oxidative stress. This system consists of two transcriptional regulators, SoxR and SoxS, that work collaboratively to upregulate the expression of genes involved in the detoxification of reactive oxygen species [73,74]. The antioxidant genes encoded by this regulon include those for enzymes such as SOD, catalase-peroxidase (KatG), and peroxiredoxin (AhpC). Collectively, these enzymes neutralize ROS generated by host cells as part of the immune response. Specifically, SOD converts superoxide radicals into molecular oxygen and hydrogen peroxide, while KatG and AhpC further convert hydrogen peroxide into water and molecular oxygen. In addition to these enzymes, *Salmonella* produces low molecular weight antioxidants such as glutathione, thioredoxin, and mycothiol, which act as electron donors to reduce free radicals formed during infection. By activating its antioxidant defense mechanisms, *Salmonella* can survive and replicate within host cells while resisting the oxidative killing effects of the host immune system.

The SoxRS regulon has potential therapeutic applications, particularly in treating diseases where oxidative stress is implicated. Harnessing the antioxidant capacity of this regulon may allow for the selective targeting of pathogenic or infected cells, thereby reducing oxidative damage to healthy tissues. Furthermore, this system could be instrumental in developing new antimicrobial agents. Many antibiotics exert their effects by generating ROS, leading to oxidative damage and cell death in bacteria. However, bacteria can develop resistance through the activation of their antioxidant systems. By targeting the Salmonella SoxRS regulon, it may be possible to create new antibiotics capable of overcoming such resistance mechanisms. Overall, exploring the antioxidant system of Salmonella presents significant potential for therapeutic applications, both in treating oxidative stress-related diseases and in developing novel antimicrobial agents.

#### 3.2.3. Typhoid fever, antibacterial therapy and oxidative stress.

Antibacterial therapy is a crucial component of typhoid fever treatment, as it helps to eliminate the bacterial infection causing the illness. However, recent studies have highlighted the potential impact of antibacterial agents on oxidative stress in patients with typhoid fever [12,75,76]. One study found that treatment with ceftriaxone, an antibacterial agent, significantly increased oxidative stress markers in rat model during treatment phase with slight decrease after treatment [75]. Another study showed that unlike in treated cases, on-treatment participants show an elevated MDA levels compared to control [12]. It was equally reported that activities of superoxide dismutase and catalase, and levels of glutathione in the tissues of treated animals were increased significantly, while MDA and nitric oxide levels were significantly decreased following treatment with anti-typhoid agents [76]. Generally, antibiotics can contribute to oxidative stress by disrupting the balance of gut bacteria [77]. The gut microbiome plays a vital role in maintaining overall health, and antibiotics can disrupt this delicate balance by killing off beneficial bacteria along with the harmful ones. This disruption can lead to an overgrowth of harmful bacteria, which can produce free radicals and contribute to oxidative stress.

Also, the dual oxidant and antioxidant effects of various antibacterial agents on oxidative stress has been documented [78], revealing that while some agents exhibit antioxidant properties, others have oxidant effects. Further research is thus needed to better understand the underlying mechanisms and potential interventions. Owing to the fact that oxidative stress and the antioxidant mechanism in typhoid cases can be both affected by antibacterial therapy, supplementation with antioxidants, including vitamin C and zinc can reduce oxidative stress and improve clinical outcomes in patients with typhoid fever.

### 3.3. Oxidative stress during *Plasmodium* & *Salmonella* coinfection

Identifying the effects of parasitic co-infection in a natural setting can be challenging as parasites can interact with each other in intricate and unpredictable ways that may not align with our perception. Oxidative stress during co-infections with *Plasmodium* and *Salmonella* species can have a significant impact on the host’s biology, including the level of antioxidant capacity. However, the specific effects of *Plasmodium* and *Salmonella* co-infection on oxidative stress can be influenced by many factors, including the species or variants involved, the host’s immune response, and other environmental factors [79]. According to a study [72], the co-infection of typhoid and malaria significantly altered the antioxidant response of infected individuals. The study recorded a significant depletion of erythrocyte GSH, an increase in plasma GSH, and increased expression of CAT in both plasma and erythrocyte. These findings suggest that co-infection with multiple pathogens can have a significant impact on an individual’s antioxidant response, potentially leading to further complications.

The specific effects of oxidative stress in malaria and typhoid co-infection are not fully understood and there is a need for mechanistic studies that can dissect the effects of oxidative stress in specific pathogenic mechanisms during malaria and typhoid fever comorbidity. Proper management and treatment of co-infections are essential in reducing the risk of oxidative stress-related complications in malaria and/or typhoid fever.

## 4. Discussion

The links between oxidative stress and immune response in malaria, typhoid fever, and their co-infections is marked by a dysregulation in oxidant-antioxidant balance. Numerous studies confirm elevated oxidative stress across patient groups, with MDA frequently cited as a key biomarker (Table 1). For instance, Abubakar et al., [23] and Kamble et al., [24] observed increased MDA and decreased vitamins A, C, E, and GSH in *P. falciparum*-infected children, highlighting lipid peroxidation and antioxidant depletion. This is consistent with findings by Krishna et al. [25], where malaria infection induced significant increases in cholesterol, LDL, triglycerides, and MDA, suggesting a metabolic-oxidative interplay.

Severe malaria presents even more pronounced oxidative disturbances. Nidhi et al., Nidhi et al., 2012 [26] reported significantly reduced antioxidant markers—ceruloplasmin, SOD, and GSH—accompanied by increased protein carbonyl and MDA. These alterations are age-dependent as shown by Olusegun [27], who found MDA increases in children aged 2–5 years, while younger infants showed compensatory increases in antioxidant enzymes. Similarly, Ezzi et al., [28] demonstrated elevated LDH and alkaline phosphatase (ALP), with concurrent declines in SOD and vitamin C among symptomatic malaria patients. Animal models reinforce these trends. Shukla et al. [29] showed co-infected mice exhibited MDA accumulation and suppressed catalase and SOD across organs. Studies by Oluba et al. [31] and Siddiqi et al., [38] further demonstrate that antimalarial treatments can restore redox balance, elevating protective enzymes like GPx, GST, and G6PDH.

Oxidative stress is also a significant factor in typhoid fever. Bayim et al., [12] found improved antioxidant levels post-treatment, including increased vitamin C and β-carotene. Also, Garba et al., [34] observed pre-surgical typhoid perforation patients had elevated MDA and disrupted SOD and catalase, linked to metal ion dysregulation. While co-infection studies illustrate compounded effects. Patel et al., [37] reported that dual infections resulted in excessive oxidative stress and liver damage, while artesunate treatment mitigated these effects. Singh et al., [39] observed that vaccination modulated oxidative enzymes, increasing GSH and reducing catalase in target organs, pointing to immune-oxidative modulation as a therapeutic mechanism.

Overall, the evidence indicates that oxidative imbalance is central to the pathology of malaria and typhoid, individually and in co-infection. Redox markers like MDA, SOD, GSH, and catalase provide insight into disease severity and therapeutic response. Antioxidant-supportive therapies may enhance recovery and mitigate organ damage in co-infected individuals.

## 5. Conclusion

Malaria and typhoid fever, though caused by distinct pathogens, share oxidative stress as a converging pathological mechanism contributing to disease progression and host tissue damage. In malaria, red blood cell hemolysis and the subsequent release of free heme provoke an immune-mediated oxidative burst. In typhoid fever, phagocytic responses to bacterial invasion can lead to ROS production that similarly promotes tissue injury. Current evidence supports the role of oxidative stress in exacerbating disease severity in both conditions, and preliminary studies suggest that antioxidant or anti-inflammatory interventions may offer therapeutic benefit.

However, the precise mechanistic pathways, optimal biomarkers for clinical monitoring, and the long-term efficacy of redox-modulating therapies remain inadequately understood. There is also limited research on oxidative dynamics in co-infected individuals, where interactions between pathogens and immune responses may amplify oxidative damage. To advance clinical management, future research should focus on standardized biomarker profiling, controlled trials evaluating antioxidant adjunct therapies, and longitudinal studies in endemic settings.

### Strength and drawbacks of this paper

This review paper focuses on elucidating the role of oxidative stress in malaria and typhoid fever comorbidity’ and can offer a comprehensive understanding of the current state of research in this area. The paper has synthesized diverse studies and provides valuable insights into the potential mechanisms underlying the association between oxidative stress and the co-occurrence of malaria and typhoid fever. The review also highlights new research directions and suggests novel therapeutic targets. Moreover, this review paper can help identify research gaps and inconsistencies, leading to a better understanding of the limitations of current knowledge and paving the way for future investigations.

However, this review paper is only as good as the quality and quantity of studies included in the analysis. The review’s conclusions will be limited by the availability and quality of research studies in the field. Moreover, bias in the selection process could skew the results of the review, leading to potential misinterpretations. Additionally, this review has generalized the findings from various studies and cannot provide the same depth of analysis as individual research studies. Therefore, caution must be taken when interpreting the results of this review paper, as they may not represent the entire scope of research on a particular topic.

## Supporting information

S1 Data PRISMA-ScR Checklist. (DOCX)

S1 File Search strategy and data extraction. (DOCX)

S2 File List of legends. (DOCX)
