# Supplementary material for: Oxidative stress in malaria and typhoid fever: A scoping review of the pathogenic mechanisms and therapeutic implications
Source: PLOS Glob Public Health. 2025 Oct 9;5(10):e0005193. doi: 10.1371/journal.pgph.0005193 (PMC12510597; doi:10.1371/journal.pgph.0005193)
Supplement: S1 File — (DOCX) [file pgph.0005193.s002.docx]

Review title: **Oxidative stress in malaria and typhoid fever: a scoping review of the pathogenic mechanisms and therapeutic implications.**

Keywords: ***Malaria, typhoid fever, oxidative stress, antioxidants, pathogenesis, therapy***

| keyword | MeSH Terms |
| --- | --- |
| ***Oxidative stress*** | Stress OR Oxidative DNA Damage OR Oxidative Damage OR Nitrosative Stress OR Nitro-Oxidative Stress OR Oxidative Nitrative Stresses OR Oxidative Injury OR Oxidative Stress Injury OR Oxidative Cleavage OR Antioxidative Stress OR Anti oxidative Stress |
| ***Antioxidant*** | Endogenous Antioxidants |
| ***Therapy*** | Therapeutic OR Treatment |
| ***Malaria*** | Plasmodium Infection OR Paludism OR Remittent Fever OR Marsh Fever |
| ***Typhoid fever*** | Salmonella typhi Infection OR Abdominal Typhus OR Enteric Fever |
| ***Pathogenesis*** | Pathogeny OR Pathogenicity OR Pathology |

**History and Search Details**

| **#** | **Search query** | **Search results** |
| --- | --- | --- |
| 1 | Search: **oxidative stress** | 347,834 |
| 2 | Search: **Oxidative stress OR Stress OR Oxidative DNA Damage OR Oxidative Damage OR Nitrosative Stress OR Nitro-Oxidative Stress OR Oxidative Nitrative Stresses OR Oxidative Injury OR Oxidative Stress Injury OR Oxidative Cleavage OR Antioxidative Stress OR Anti oxidative Stress** | 1,410,409 |
| 3 | Search: **Antioxidant OR Endogenous Antioxidants** | 773,371 |
| 4 | Search: **Therapy OR Therapeutic OR Treatment** | 14,560,006 |
| 5 | Search: **Malaria OR Plasmodium Infection OR Paludism OR Remittent Fever OR Marsh Fever** | 118,388 |
| 6 | Search: **Typhoid fever OR Salmonella typhi Infection OR Abdominal Typhus OR Enteric Fever** | 17,561 |
| 7 | Search: **Pathogenesis OR Pathogeny OR Pathogenicity OR Pathology** | 13,181,333 |
| 8 | Search: **(Malaria OR Plasmodium Infection OR Paludism OR Remittent Fever OR Marsh Fever) AND (Oxidative stress OR Stress OR Oxidative DNA Damage OR Oxidative Damage OR Nitrosative Stress OR Nitro-Oxidative Stress OR Oxidative Nitrative Stresses OR Oxidative Injury OR Oxidative Stress Injury OR Oxidative Cleavage OR Antioxidative Stress OR Anti oxidative Stress)** | 2,073 |
| 9 | Search: **((Typhoid fever OR Salmonella typhi Infection OR Abdominal Typhus OR Enteric Fever) AND (Oxidative stress OR Stress OR Oxidative DNA Damage OR Oxidative Damage OR Nitrosative Stress OR Nitro-Oxidative Stress OR Oxidative Nitrative Stresses OR Oxidative Injury OR Oxidative Stress Injury OR Oxidative Cleavage OR Antioxidative Stress OR Anti oxidative Stress)) OR (Antioxidant OR Endogenous Antioxidants)** | 773,506 |
| 10 | Search: **(((Typhoid fever OR Salmonella typhi Infection OR Abdominal Typhus OR Enteric Fever) AND (Oxidative stress OR Stress OR Oxidative DNA Damage OR Oxidative Damage OR Nitrosative Stress OR Nitro-Oxidative Stress OR Oxidative Nitrative Stresses OR Oxidative Injury OR Oxidative Stress Injury OR Oxidative Cleavage OR Antioxidative Stress OR Anti oxidative Stress)) OR (Antioxidant OR Endogenous Antioxidants)) AND ((Malaria OR Plasmodium Infection OR Paludism OR Remittent Fever OR Marsh Fever) AND (Oxidative stress OR Stress OR Oxidative DNA Damage OR Oxidative Damage OR Nitrosative Stress OR Nitro-Oxidative Stress OR Oxidative Nitrative Stresses OR Oxidative Injury OR Oxidative Stress Injury OR Oxidative Cleavage OR Antioxidative Stress OR Anti oxidative Stress))** | 467 |
| 11 | Search: **((((Typhoid fever OR Salmonella typhi Infection OR Abdominal Typhus OR Enteric Fever) AND (Oxidative stress OR Stress OR Oxidative DNA Damage OR Oxidative Damage OR Nitrosative Stress OR Nitro-Oxidative Stress OR Oxidative Nitrative Stresses OR Oxidative Injury OR Oxidative Stress Injury OR Oxidative Cleavage OR Antioxidative Stress OR Anti oxidative Stress)) OR (Antioxidant OR Endogenous Antioxidants)) AND ((Malaria OR Plasmodium Infection OR Paludism OR Remittent Fever OR Marsh Fever) AND (Oxidative stress OR Stress OR Oxidative DNA Damage OR Oxidative Damage OR Nitrosative Stress OR Nitro-Oxidative Stress OR Oxidative Nitrative Stresses OR Oxidative Injury OR Oxidative Stress Injury OR Oxidative Cleavage OR Antioxidative Stress OR Anti oxidative Stress))) AND (Therapy OR Therapeutic OR Treatment)** | 298 |
| 12 | Search: **(((((Typhoid fever OR Salmonella typhi Infection OR Abdominal Typhus OR Enteric Fever) AND (Oxidative stress OR Stress OR Oxidative DNA Damage OR Oxidative Damage OR Nitrosative Stress OR Nitro-Oxidative Stress OR Oxidative Nitrative Stresses OR Oxidative Injury OR Oxidative Stress Injury OR Oxidative Cleavage OR Antioxidative Stress OR Anti oxidative Stress)) OR (Antioxidant OR Endogenous Antioxidants)) AND ((Malaria OR Plasmodium Infection OR Paludism OR Remittent Fever OR Marsh Fever) AND (Oxidative stress OR Stress OR Oxidative DNA Damage OR Oxidative Damage OR Nitrosative Stress OR Nitro-Oxidative Stress OR Oxidative Nitrative Stresses OR Oxidative Injury OR Oxidative Stress Injury OR Oxidative Cleavage OR Antioxidative Stress OR Anti oxidative Stress))) AND (Therapy OR Therapeutic OR Treatment)) AND (Pathogenesis OR Pathogeny OR Pathogenicity OR Pathology)** | 179 |
| 13 | Search: **(((((Typhoid fever OR Salmonella typhi Infection OR Abdominal Typhus OR Enteric Fever) AND (Oxidative stress OR Stress OR Oxidative DNA Damage OR Oxidative Damage OR Nitrosative Stress OR Nitro-Oxidative Stress OR Oxidative Nitrative Stresses OR Oxidative Injury OR Oxidative Stress Injury OR Oxidative Cleavage OR Antioxidative Stress OR Anti oxidative Stress)) OR (Antioxidant OR Endogenous Antioxidants)) AND ((Malaria OR Plasmodium Infection OR Paludism OR Remittent Fever OR Marsh Fever) AND (Oxidative stress OR Stress OR Oxidative DNA Damage OR Oxidative Damage OR Nitrosative Stress OR Nitro-Oxidative Stress OR Oxidative Nitrative Stresses OR Oxidative Injury OR Oxidative Stress Injury OR Oxidative Cleavage OR Antioxidative Stress OR Anti oxidative Stress))) AND (Therapy OR Therapeutic OR Treatment)) AND (Pathogenesis OR Pathogeny OR Pathogenicity OR Pathology)** Filters: **English, Exclude preprints, from 2000 - 2024** | 158 |
|  |  |  |
