# Supplementary material for: Oxidative stress in malaria and typhoid fever: A scoping review of the pathogenic mechanisms and therapeutic implications
Source: PLOS Glob Public Health. 2025 Oct 9;5(10):e0005193. doi: 10.1371/journal.pgph.0005193 (PMC12510597; doi:10.1371/journal.pgph.0005193)
Supplement: S2 File — (DOCX) [file pgph.0005193.s003.docx]

This document outlines the comprehensive search strategy implemented for the scoping review titled "**Oxidative stress in malaria and typhoid fever: a scoping review of the pathogenic mechanisms and therapeutic implications**." The content includes the following elements:

**1. Keywords:** These are core terms identified as central to the review topic including 'oxidative stress', 'antioxidants', 'malaria', 'typhoid fever', 'pathogenesis', and 'therapy'.

**2. MeSH terms (Medical Subject Headings):** Controlled vocabulary terms used to ensure standardized indexing in databases. For instance, terms like 'Oxidative DNA Damage', 'Nitrosative Stress', and 'Antioxidative Stress' ensure inclusivity of related concepts.

**3. Search queries:** This section documents the exact Boolean logic used in various combinations for database searches. Examples include:

- Oxidative stress OR Stress OR Oxidative DNA Damage

- Malaria OR Plasmodium Infection OR Marsh Fever

- Typhoid fever OR Salmonella typhi Infection OR Enteric Fever

- Combined queries with AND operators to intersect terms (e.g., malaria AND oxidative Stress)

**4. Search results:** Each search query is accompanied by the number of hits retrieved from the database, illustrating the scope and relevance of each combination. For example, searching for 'Antioxidant OR Endogenous Antioxidants' yielded over 770,000 results.

**5. Search filters and limits:** The final advanced queries applied filters such as language (English), exclusion of preprints, and a publication date range from 2000 to 2024.

This structured search strategy supports the transparency and reproducibility of the review’s methodology.
